# The impact of parameter variation in the quantification of forensic genetic evidence

**DOI:** 10.1038/s41598-024-83841-2

**Published:** 2025-01-20

**Authors:** Camila Costa, Carolina Figueiredo, Sandra Costa, Paulo Miguel Ferreira, António Amorim, Lourdes Prieto, Nádia Pinto

**Affiliations:** 1 Departamento de Biologia, Faculdade de Ciências, Universidade do Porto, Porto, Portugal; 2 i3S - Instituto de Investigação e Inovação em Saúde, Universidade do Porto, Porto, Portugal; 3 Biologia, Laboratório de Polícia Científica da Polícia Judiciaria (LPC-PJ), Lisbon, Portugal; 4 IPATIMUP - Instituto de Patologia e Imunologia Molecular da Universidade do Porto, Porto, Portugal; 5 Grupo de Medicina Xenómica, Instituto de Ciencias Forenses, Universidad de Santiago de Compostela, Santiago de Compostela, Spain; 6 Laboratorio ADN, Comisaría General de Policía Científica, Madrid, Spain; 7 CMUP, Centro de Matemática da Universidade do Porto, Porto, Portugal

**Keywords:** Forensic DNA, Mixture samples, Likelihood ratio, Drop-in, Analytical threshold, Stutters, Population genetics, Statistics

## Abstract

Technological advancements have allowed the detection of increasingly complex forensic genetics samples, as minimum amounts of DNA can now be detected in crime scenes or other settings of interest. The weight of the evidence depends on several parameters regarding the population and sample-related analytical factors, the latter in a greater number when the DNA amount is considered. This led to the development of probabilistic genotyping software (PGS), able to deal with the associated complexities. This study aims to evaluate the impact on the evidence’s weighing, when different analytical threshold values are used, and when different models and/or estimates for analytical artifacts, such as stutters or drop-in parameters, are considered. To reach this goal, three PGS, based on different statistical models, were used to analyze real casework pairs of samples composed of a mixture with either two or three estimated contributors, and a single-source sample associated. The obtained results show that the estimation of these parameters must not be overlooked, as they may considerably impact the outcome. This underlines the importance of proper parametrization in the analysis of forensic genetics identification problems when using complex samples, and the understanding by practitioners of how probabilistic genotyping informatics tools work to use them accurately.

## Introduction

With the urge to overcome the difficulty and subjectivity of interpreting complex DNA casework samples, probabilistic genotyping methods were developed and implemented as software. These software tools allow the experts to quantify the weight of the genetic evidence with a robust and scientifically supported statistical approach, considering the short tandem repeat (STR) profiles of evidence and reference(s) samples obtained through the gold standard genotyping methodology of fragment length determination—capillary electrophoresis (CE).

DNA samples recovered from crime scenes may be composed of contributions from more than one donor—DNA mixture—and may have several features that make them complex and difficult to analyze, as described next. The decision on the presence (or not) of non-allelic peaks (artifacts or stochastic effects)^1–10^ is a crucial step for the quantification of the evidence, as well as the estimation of the number of contributors to the mixture, both depending on the evaluation of the expert^11–16^. Besides the true number of contributors being always unknown and having to be estimated by the expert, other constraints make these samples’ interpretation difficult, such as a. allele sharing between contributors, b. contributions in different proportions, c. stutter peaks which may be confused with alleles of a minor contributor (or vice versa), or even d. amplification stochastic effects (heterozygotic imbalance, drop-in, and/or drop-out) due to low-template DNA.

Indeed, despite the important role of computer programs in evaluating the evidence, the expert continues to have a crucial role in the entire course regarding the weight of the evidence^17^. It is the expert’s responsibility to perform a correct and meticulous analysis from the very beginning of the process, that is, from the evidence collection to the electropherogram (EPG) analysis, so that possible errors are minimized. Also, internal protocols based on international and well-established guidelines should be strictly followed to avoid bias and maintain consistency between and within the analysis. A well-collected sample, i.e. maximizing the genetic material subjected to analysis and minimizing the possible sources of contamination, provides a profile easier to interpret, reducing ambiguity and therefore minimizing erroneous conclusions^18–22^. The STR profile is interpreted by the expert through the EPG analysis after sample processing—that is, after the DNA extraction, purification, quantification, and amplification, followed by STR separation and detection, and finally STR genotyping.

Once the genotypic profiles (of both the unknown and corresponding reference sample) are obtained and pre-processed, the weight of the DNA evidence may be quantified in probabilistic genotyping software^23,24^. The weight of the evidence is performed through the computation of a Likelihood Ratio (LR) value^25,26^, which provides a comparison between the probability of observing the genetic evidence assuming two alternative and mutually exclusive hypotheses. In standard identification problems, these hypotheses are “H1 = The person of interest (POI) is a contributor of the mixture” and, alternatively, “H2 = The POI is not a contributor nor genetically related to any contributor of the mixture”. This calculation depends on several parameters regarding population—alleles frequencies and coancestry coefficient—and analytical factors—drop-in, dropout, analytical threshold, and stutters, which will be described next.

The probabilistic genotyping tools are based on different models regarding the EPG information considered to quantify the genetic evidence^27,28^. If only the qualitative information (observed alleles in the profile) is considered, software are said to be based on a qualitative, or semi-continuous, model—e.g., Lab Retriever^29^ or LRmix Studio^30^. On the other hand, if both qualitative (observed alleles) and quantitative information (height of the observed allele peaks) are considered for computations, the software is said to be based on a quantitative, or continuous, model—e.g., EuroForMix^31^ or STRmix™^32^.

It was already shown that different informatics implementations and statistical modeling may lead to important differences in the quantification of the evidence’s weight, through both mocked and real casework samples^33–36^. Specifically in Costa et al.^17^ a comparison between LRs assigned by two quantitative (EurForMix and STRmix™) and one qualitative (LRmix Studio) informatics tools, for the same real casework input data, was performed maintaining the values/conditions of the software parameters as similar as possible. In some cases, relevant differences were observed. These results were the motto for the present work where the differences obtained in the statistical LR outputs when considering different parameters’ values for the same informatics tools are assessed. A recent study^37^ has shown that differences due to validated parameters’ conditions are small for a particular software and, in most cases, did not produce contradictory interpretations. Nevertheless, it will be interesting to understand the impact of applying different statistical models to the same parameter like the following ones considering different informatics tools.

### Drop-in and drop-out

A drop-in allele corresponds to a spurious peak observed in the sample profile, i.e., an allele that shows up in the evidence profile that resulted from a source unassociated with the sample (for example from a crime scene environment or laboratory plasticware) and, therefore, cannot be explained by any contributor. It can be distinguished from gross contamination due to a large amount of peaks detected in the latter^7,25^. This stochastic effect generates thus discordances between the mixture sample and the contributor profiles, which can lead to misinterpretations and, consequently, wrong conclusions^8,9^.

The drop-in frequency is laboratory-specific and is usually estimated considering the number of negative controls out of all negative controls in which an additional allelic component, an irreproducible and unexplained peak, appears in the EPG. A negative control is an additional reaction not containing any type of biological material (besides those of the DNA extraction, amplification reaction, and injection) that is analyzed at the same time and under the same conditions as the samples^38^. The higher the drop-in frequency, the less likely it is that an allele is considered to belong to a mixture contributor. So, the software for statistical analysis of mixture samples may consider the drop-in frequency value (in both quantitative and qualitative models) and the distribution of drop-in parameters (only in quantitative models, those considering peak heights) when computing LR values^39–41^. The information on the height of the drop-in peaks is used to estimate the distribution of drop-in parameters, which can be modeled by different statistical distributions depending on the software tool^42–44^. For example, EuroForMix models the drop-in through a lambda distribution (λ), while STRmix™ employs either a gamma (ɣ) or a uniform distribution. If limited observations of drop-in have been made or if the dataset is limited in size, the STRmix™ may not be able to develop a reliable gamma model. So, instead of determining the parameters of the gamma distribution (ɑ and β), the use of a uniform distribution is recommended.

Conversely, allele dropout is a stochastic amplification event that causes an allele present in the contributors’ profiles to be absent from the mixture profile to which they contributed due to the low quality and quantity of DNA usually encountered in forensic samples^7^. As for drop-in, discordances between the mixture sample and the contributor profiles may be generated, and the proper modeling of the dropout phenomena should also be considered for an accurate analysis^8,9^. Parameters related to the dropout probability may have to be estimated and introduced by the user as in qualitative tools^30^ or could be directly estimated by the software through the height of the peaks in the case of quantitative programs^26,39–41,44–47^. The latter will model this parameter with the same function used to model the peak height distribution, which can differ between tools. For example, EuroForMix software models peak height using a gamma distribution, while STRmix™ uses a log-normal distribution^31^. As there has been an increasing use of quantitative tools, under which drop-out estimates are automatically calculated directly by the software with no possible user action, this parameter will not be further analyzed in this study.

### Analytical threshold

The analytical threshold is a previously defined value (measured in relative fluorescence units, RFUs) introduced in the peak detection software, for distinguishing true alleles from the baseline noise, considering only as true alleles peaks higher than the defined threshold. The baseline noise consists of non-reproducible peaks, resulting from electronic fluctuations, air bubbles, and crystals in the polymer in CE capillary, or low-level sample contamination^5^. There is no standard rule for determining the analytical threshold value that should be used, and each laboratory should establish its values based on the corresponding internal validation procedures. However, it should always be taken into account that the estimation of this value encloses a risk-reward decision. Indeed, a value that is too high may result in loss of information due to the discarding of true alleles with smaller heights, substantially affecting the global LR computed value. On the other hand, a value that is too low may result in the incorrect assignment of the peaks, that is, baseline noise peaks being considered true alleles. In any case, it should be reinforced that the determination of this analytical threshold must be part of the internal validation procedure of any laboratory, which may depend on several factors including those related to the type of samples to be analyzed^48^. There are several methods available to assist experts in the estimation of this parameter, as is the case of STR-validator (free and open-source software available at https://sites.google.com/site/forensicapps/strvalidator) that can also be used to estimate other parameters^49^.

Quantitative probabilistic genotyping tools incorporate this parameter since they consider alleles’ peak height to perform their computations. In contrast, in qualitative tools, any detected peak is considered a true allele.

### Stutter artifacts

Stutter peaks are a PCR product artifact that results from a slipped-strand mispairing during the PCR extension phase and constitutes the most encountered artifact in an EPG. When the two strands (template and extending strand) re-anneal, one of them loops out and the other aligns in front of the supposed position. If the loop occurs in the template strand one or more repeat units are deleted in the new strand, but if the loop happens in the extending strand it will result in the addition of repeat(s) unit(s) to the new strand (less common). Considering single-source samples, this artifact can be easily identified by its size and position; however, when analyzing mixed-source samples, it becomes difficult to differentiate it from a minor contributor allele^1,2^. To maximize the likelihood that no true allele information is lost, various studies advocate the use of software able to model stutters when analyzing the evidence sample instead of either using a stutter filter (during the STR genotyping phase)^3^ or relying on (subjective) human decisions^4,5^. Based on these studies, quantitative software such as STRmix™ requires the inclusion of stutters in the evidence sample^50^. On the other hand, other quantitative tools—e.g., EuroForMix—give the user the possibility to decide whether or not to include several types of stutters as an extension of the model^31^. STRmix™ models locus-specific amplification efficiencies and considers expected stutter ratios per locus defined using empirical data. EuroForMix does not model locus-specific amplification efficiencies and uses instead a blanket expected stutter rate^44^. As far as qualitative software are concerned, for tools like LRmix Studio, the inclusion of stutters is not recommended and peak stutters should be removed from mixture samples before analysis^30^.

### Goal of this study

The specific aim of this work is to demonstrate the impact that considering different values and/or conditions for the software parameters related to drop-in, analytical threshold, and stutters may have on the quantification of the evidence. Considering software based in both qualitative—LRmix Studio v.2.1.3^30^—and quantitative models—EuroForMix v.3.4.0^31^ and STRmix™ v.2.7^32^, computations were performed using real casework samples and the obtained LR values were compared in an intra-software analysis when parameter values/conditions were varied.

The major objective of this work is to make practitioners aware of the importance of accurate estimation and modeling of the parameters that influence the weight of the evidence, as variations of these may have an important impact in forensic genetics routine analyses.

## Material and methods

Resorting to evidence material from former cases of the Portuguese Scientific Police Laboratory, Judiciary Police, 154 irreversibly anonymized DNA mixtures with two and three previously estimated contributors, equally distributed, were selected. In addition, 154 single-source samples, each one related to the forensic cases where the DNA mixtures were detected, were also considered. Some of the single-source samples were real reference samples collected from known individuals (POI, either victim or suspect, e.g.); but some others were single-source evidence profiles detected at the same crime scene where the DNA mixtures were also found (recovered from personal belongings of interest, as a toothbrush or a razor, e.g.). For simplification purposes, both types of single-source samples will be named “reference samples” throughout the paper. For most of the pairs, the donor of the reference sample could not be excluded from being one of the mixture contributors. All methods were carried out in accordance with relevant guidelines and regulations. The samples were previously processed in the context of the respective casework, under manufacturer and internal protocols. Evidence samples were amplified using GlobalFiler™ PCR Amplification Kit and references were amplified by using GlobalFiler™ Express PCR Amplification Kit (Applied Biosystems™), both 24-locus STR kits; and, the PCR products were detected and separated by capillary electrophoresis in 3500 Genetic Analyzer with GeneMapper™ ID-X (Applied Biosystems™), with an analytical threshold equal to 100 RFU for all the dye channels.

To develop this work, the genetic information obtained for each anonymized pair of samples (mixture/reference) analyzed for a set of 21 autosomal STR markers was considered. Real casework samples were selected for the development of this work to mimic as much as possible the conditions that forensic geneticists encountered in routine analyses, as there are levels of complexity that cannot be anticipated in mocked samples. Most of the analyzed genotypic data coincide with those of Costa et al.^17^. Proper ethics approval was obtained from the Committee for Ethical and Responsible Conduct of Research of i3S—Instituto de Investigação e Inovação em Saúde, Universidade do Porto, Portugal (5/CECRI/2022), regarding the statistical analysis of both environmental and reference genetic samples (fully and irreversibly anonymized, when applied). For confidentiality reasons, the EPGs or corresponding data input files of these samples cannot be made publicly available. All the statistics obtained per software and parameter for each pair of samples are available in Supplementary Tables—please see the section ‘Data Availability’ for more information regarding data sharing.

For each pair of samples, an LR assuming the alternative hypotheses: “The POI is a contributor of the mixture” and “The POI is not a contributor nor genetically related to any contributor of the mixture” was calculated. The reference sample was assumed to belong to the POI. The LR values were computed through both qualitative (LRmix Studio v.2.1.3^30^) and quantitative (EuroForMix v.3.4.0^31^ and STRmix™ v.2.7^32^) software tools. In EuroForMix, the Maximum Likelihood Estimation (MLE) approach was employed as suggested by software developers for model comparison purposes^51^. Different software tools were considered to analyze the impact of the variation of each parameter under alternative statistical and computational frameworks and architectures. For all the computations performed the coancestry coefficient of 0.01 was considered, as recommended by the second National Research Council report recommended for general populations^52^; as well as the allele frequencies of the National Institute of Standards and Technology (NIST) database concerning the Caucasian population^53^—see Supplementary Table S1.

When computing the LR values using the above-mentioned tools, distinct parameter values were introduced by the user, related to the Drop-in (Frequency and Modeling), Analytical Threshold, and Stutters.

For each one of the parameters, “default” and “varied” values were defined as detailed in Table 1 to quantify the impact the different parameter values may have on LR computations. The parameters’ values set as “default” were either predefined in the software or presented in the literature, while the “varied” values were sub- and/or over-variations of the default ones. The rationale for the different values established is presented in the following subsections. In any case, it should be remarked that most of the parameter values predefined in the different software should be optimized considering each laboratory’s data. The parameter values considered in this work are for comparison purposes.

**Table 1:** Default and varied values introduced in LRmix Studio v.2.1.3, EuroForMix v.3.4.0, and STRmix™ v.2.7 for each analyzed parameter.

Parameters	Values					
	LRmix studio		EuroForMix		STRmix™	
	Default	Variation	Default	Variation	Default	Variation
Drop-in frequency	0.05	0 0.1	0.05	0 0.1	0.05	0 0.1
Drop-in parameters’ distribution	N/A	N/A	λ: 0.01	λ: 0.05	ɣ: (1.0,1.0)	Uniform
Analytical threshold	N/A	N/A	100	150	100	150
Stutters	Without	N/A	With	Without	With	N/A

N/A: not applicable.

To verify the effect that each parameter—drop-in, analytical threshold, and stutters—may have on the weight of the evidence, LR values were computed varying only one parameter at a time, as outlined in Supplementary Table S2. As it is the standard practice in forensic routine, in this work is considered a single computation for each software, pair of samples, and set of parameter values. For reseach purposes, exceptions to this procedure are described next. Whenever an LR equal to zero was obtained for a specific marker, under the parameters’ default values, the marker was removed from all the analyses considering that specific pair of samples and software. For these cases, the LR value provided in Supplementary Table S3 is denoted as LR_<21_, as less than 21 markers were considered. For 17% and 9% of the cases with mixtures with two and three estimated contributors, respectively, STRmix™ returned an LR equal to zero. In these cases, either alleles present in the single contributor sample were absent from the mixture sample, or Markov Chain Monte Carlo (MCMC) sampling failed to identify the true genotype combination, i.e., MCMC algorithms failed to converge, as similarly observed in other studies^36,44,54^. To illustrate the stochasticity of the architecture of the STRmix™ tool and how this may affect the results presented in this study, corresponding computations were also replicated for the pair of samples showing the maximum LR differences after parameter variation. This case is specifically referred to and analyzed in the text. For all the STRmix™ computations, the MCMC-related parameters provided by default in the software were those considered—Burn-in accepts per chain of 10,000 and Post burn-in accepts per chain of 50,000.

Then, for each analyzed parameter, an intra-software analysis was carried out through a comparison of the LR values computed assuming the default (LR1) and varied conditions (LR2) for each pair of samples through the computation of their ratio R = LR1/LR2 if LR1 > LR2, or R = LR2/LR1, otherwise. Thus, for example, 1 < R < 10 indicates that one of the LRs (either computed assuming the default or varied condition) is at most ten times greater than the other. Afterward, a statistical test was performed to infer a significant increasing or decreasing trend (ɑ = 0.05) regarding the LR values that were observed when the parameter value was or not varied in each statistical and informatics framework.

When for certain parameters’ values the software couldn’t compute an LR value different from zero—i.e. either computing an LR equal to zero or presenting an error message—no comparisons were performed for those cases.

### Drop-in and drop-out

Remarking that drop-in parameters are laboratory-specific, some values were established due to operational reasons for proceeding with the study. The drop-in frequency was then set to 0.05 as default, a value considered reasonable in similar studies^55^, also coinciding with the pre-set value in LRmix Studio. The variations considered were: (a) 0, meaning that alleles not attributed to a contributor cannot be considered as a drop-in peak; and (b) 0.1, a high probability of unexplained alleles being a drop-in (the double of the determined default value). Also related to drop-in modeling, the impact of considering variations for the distribution of their heights was tested in the quantitative computer tools, which is computed differently in EuroForMix and STRmix™. In EuroForMix the drop-in is modeled using a lambda distribution—the value of the hyperparam λ equal to 0.01 was used as default (which also corresponds to the software’s default value) and 0.05 as a varied value; λ cannot be equal to zero. On the other hand, STRmix™ employs either a gamma (ɣ) or uniform distribution. Therefore, both distributions were tested. The gamma distribution was considered the “default” condition, for which the software default values of parameters ɑ and β were used (1.0 and 1.0, respectively), while uniform distribution was considered the “varied” condition. Regarding Drop-out, and as previously mentioned, it is directly estimated through the peak height distribution of the alleles in the quantitative tools (EuroForMix and STRmix™), while it was maintained the default value equal to 0.1 in the qualitative tool (LRmix Studio).

### Analytical threshold

The analytical threshold is a parameter required only in quantitative tools—EuroForMix and STRmix™. This parameter value must be the same as used in the software from which the sample files are exported. In this case, 100 RFU was defined as the default and 150 RFU as the varied value.

### Stutter artifacts

The presence/absence of stutters, and corresponding modeling, in mixture sample profiles were also tested. In EuroForMix analysis, modeling of stutters is possible but not required, and so, LR values computed for each pair with (default) and without stutters (varied condition) in mixture samples profiles were compared. In the first condition, EPG data with unfiltered stutters were considered and both backward (N − 1) and forward (N + 1) stutters were modeled; in the second condition, EPG data with filtered stutters were considered. On the other hand, neither in LRmix Studio nor in STRmix™ was possible to perform this evaluation due to opposite reasons. In LRmix Studio, the developers recommend filtering stutters previously, while in STRmix™ its inclusion is mandatory and its estimation is directly computed by the software.

## Results and discussion

The LR values for each one of the 154 pairs of samples, comprising one mixture (with two or three estimated contributors) and a reference sample that in most of the cases cannot be excluded as contributor, were computed using the three software: LRmix Studio v.2.1.3^30^, EuroForMix v.3.4.0^31^, and STRmix™ v.2.7^32^, the first considering only the presence/absence of alleles, and the other two also the DNA quantity. LR values were computed under both default and varied conditions for each parameter—see Supplementary Tables S2 and S3.

### Drop-in

#### Frequency

The frequency of drop-in was varied from a null value to the double (0.1) of the considered default value (0.05), as summarily previously presented^56^.

Table 2 shows the differences found between the LR values computed assuming the default and the varied conditions, for both cases with mixtures with two and three estimated contributors. These differences are presented under the format of a ratio between the computed LRs.

**Table 2:** Ratio R (R > 1) between LR values computed considering the varied values of drop-in frequency (0 and 0.1) and the default value (0.05) by LRmix Studio v.2.1.3, EuroForMix v.3.4.0, and STRmix™ v.2.7, for both cases with mixtures with two and three estimated contributors.

Estimated number of contributors						
2						
Ratio between LRs (default vs varied, R > 1)	LRmix studio		EuroForMix		STRmix™	
	Drop-in frequency		Drop-in frequency		Drop-in frequency	
	0.05 vs. 0	0.05 vs 0.1	0.05 vs. 0	0.05 vs 0.1	0.05 vs. 0	0.05 vs 0.1
1 < R < 10	74%	100%	87%	100%	92%	95%
10 < R < 100	0%	0%	4%	0%	4%	4%
100 < R < 1,000	0%	0%	4%	0%	4%	0%
1,000 < R < 10,000	0%	0%	1%	0%	0%	1%
R > 10,000	0%	0%	1%	0%	0%	0%
N/A	26%	0%	3%	0%	0%	0%
Maximum ratio between LR values						
R	1.20E+00	8.09E+00	8.50E+10	1.90E+00	8.83E+02	3.05E+03
LR (default)	2.20E+13	1.83E+03	2.71E+23	2.78E+08	3.91E+24	5.58E+14
LR (varied)	2.64E+13	1.48E+04	3.19E+12	5.29E+08	4.43E+21	1.70E+18
Pair*	53_2	1_2	6_2	2_2	51_2	8_2
LR tends to (α = 0.05)						
	Increase	Decrease	Increase	Decrease	–	–
p-value	5.62E−08	6.65E−05	1.82E−03	2.48E−05	2.10E−01	1.38E−01

3						
Ratio between LRs (default vs varied, R > 1)	LRmix studio		EuroForMix		STRmix™	
	Drop-in frequency		Drop-in frequency		Drop-in frequency	
	0.05 vs. 0	0.05 vs 0.1	0.05 vs. 0	0.05 vs 0.1	0.05 vs. 0	0.05 vs 0.1
1 < R < 10	96%	100%	97%	100%	84%	86%
10 < R < 100	0%	0%	1%	0%	9%	8%
100 < R < 1,000	0%	0%	0%	0%	3%	3%
1,000 < R < 10,000	0%	0%	0%	0%	1%	0%
R > 10,000	0%	0%	1%	0%	3%	3%
N/A	4%	0%	1%	0%	0%	1%
Maximum ratio between LRs						
R	1.18E+00	2.07E+00	1.17E+12	1.81E+00	7.86E+08	4.24E+06
LR (default)	3.31E+04	1.23E+07	4.61E+09	1.77E+09	1.51E+04	1.12E+17
LR (varied)	2.81E+04	2.54E+07	5.40E+21	3.20E+09	1.92E-05	4.75E+23
Pair*	18_3	4_3	5_3	8_3	9_3	49_3
LR tends to (α = 0.05)						
	Increase	Decrease	Increase	Decrease	–	–
p-value	1.99E−04	4.11E−04	2.86E−03	9.50E−04	8.19E−01	3.59E−01

N/A: “Not Applicable” corresponds to situations where no comparisons were performed, either because an LR equal to zero was computed, or an error message was displayed. The evidence of a general trend for the LR values either increasing or decreasing when the drop-in frequency was varied is presented along with the corresponding p-value (α = 0.05); significant values are underlined. *See Tables S3_1 in Supplementary Table S3 for more data.

Through the analysis of computed LR values, a statistically significant trend (α = 0.05) for LR values increase with the decrease of drop-in frequency (both when considering 0.05 to 0, and 0.1 to 0.05) was observed for LRmix Studio and EuroForMix, for both two and three estimated contributors cases. On the other hand, no statistically significant trend towards an increase or decrease in the LR values was observed for STRmix™ (Table 2).

In general, varying the drop-in frequency resulted in LR values diverging less than ten times in most of the cases for which a non-null value was retrieved (74% to 100%). Differences greater than 10,000 times were found for some cases (six, two of them in EuroforMix) with two and/or three estimated contributors when considering quantitative tools. In four of these cases, differences were observed when disregarding drop-in frequency, but in two of them, it occurred when the estimated frequency doubled from 0.05 to 0.1. In these cases showing greater differences (R > 10,000) it is noteworthy that when the drop-in frequency was not considered in EuroForMix computation, the LR value followed an opposite trend of the generally showed by the overall results—that is, when the drop-in frequency decreased LRs weakened. On the other hand, it cannot be disregarded the stochasticity of the methods on which STRmix™ is based since it may imply that the observed differences are due to the lack of convergence of MCMC algorithms, rather than due to the parameter variation itself. Indeed, this hypothesis was explored and confirmed for the case showing the greatest differences in the LR values—obtained with default and varied parameters for drop-in modeling, see “Statistical modeling” section).

Besides the differences computed, another factor that demonstrates the impact of the parameter variation is revealed when the software cannot compute an LR value (different from zero), and, therefore, it is not possible to perform a comparison. This situation was observed in all software when comparing LR values computed assuming a null and the default (0.05) drop-in frequencies. The software computed either an LR equal to zero—as STRmix™—or does not compute a value at all—as LRmix Studio and EuroForMix. For the latter, it was observed in both cases with mixtures with two and three estimated contributors, while in STRmix™ it was only verified for cases with mixtures with three. When the drop-in frequency is considered null, the probability of drop-in peaks being treated by software as true peak alleles increases. So, these situations may have resulted from cases where the maximum number of peaks may have been exceeded considering the number of contributors estimated. Even when the number of peaks has not been exceeded, the software can infer, based on the relative height of the peaks, that more alleles are presented than would be expected, considering the number of contributors. Conversely, when the drop-in frequency value was doubled, those situations were only verified in STRmix™ for a case with a mixture with three estimated contributors. Doubling the drop-in frequency value led to an increase in the probability of some peaks being considered drop-in. Therefore, if those peaks belong to the reference sample under analysis, the LR value decreases as observed in STRmix™ computations.

In any case, the most striking results in this regard were found for the qualitative software for 26% and 4% of the cases with two and three estimated contributors, respectively, for which no LR values were retrieved when the drop-in event was disregarded.

#### Statistical modeling

As previously referred, it should be noted that, even though both software are quantitative, the drop-in modeling is done differently. In EuroForMix the drop-in is modeled using a Lambda distribution (λ) while STRmix™ uses either a Gamma (γ) or a Uniform distribution.

The differences between LR values computed assuming the default condition (λ = 0.01 for EuroForMix and γ = (1.0, 1.0) for STRmix™) and the varied condition (λ = 0.05 and Uniform, respectively), for both cases with mixtures with two and three estimated contributors, are summarized in Table 3. A possible trend for LR increase or decrease when this parameter was varied is also presented.

**Table 3:** Ratio R (R > 1) between LR values computed in EuroForMix v.3.4.0 under the default and varied drop-in condition (λ = 0.01 and 0.05, respectively) and in STRmix™ v.2.7 under the default and varied condition (γ = (1.0, 1.0) and Uniform, respectively), for cases with mixtures with two and three estimated contributors.

Ratio between LRs (default vs varied, R > 1)	Estimated number of contributors			
	2		3	
	EuroForMix	STRmix™	EuroForMix	STRmix™
	λ = 0.01 vs 0.05	γ = (1.0, 1.0) vs uniform	λ = 0.01 vs 0.05	γ = (1.0, 1.0) vs uniform
1 < R < 10	100%	94%	100%	83%
10 < R < 100	0%	4%	0%	9%
100 < R < 1000	0%	0%	0%	4%
1000 < R < 10,000	0%	0%	0%	1%
R > 10,000	0%	3%	0%	3%
Maximum ratio between LRs				
R	2.88E+00	2.12E+17	1.94E+00	3.46E+14
LR (default)	1.44E+18	3.80E+09	4.69E+23	6.53E+07
LR (varied)	4.14E+18	1.79E−08	9.10E+23	1.89E−07
Pair*	7_2	13_2^#^	64_3	8_3
LR tends to (α = 0.05)				
	–	–	Increase	–
p-value	5.27E−02	4.25E−01	3.04E−02	9.09E−01

The evidence of a general trend for the LR values increasing or decreasing when the drop-in parameter distribution was varied is presented along with the corresponding p-value (α = 0.05) that supported a trend or the absence of a trend is provided; significant values are underlined. *See Tables S3_2 in Supplementary Table S3. ^#^See the main text and Supplementary Table S4 for more details.

Only EuroForMix showed a statistically significant trend, as for cases with mixtures with three estimated contributors, the LR values tended to increase when the lambda value was varied from 0.01 to 0.05 (Table 3). Nevertheless, all differences observed in EuroForMix were lower than ten times, revealing a lower impact than those observed in STRmix™ when varying the corresponding parameters.

Although most of the computed differences in STRmix™ were also lower than ten times, differences higher than 10,000 times were observed. For those cases, the LR values decreased. In fact, the LR values dropped below one, favoring the alternative hypothesis, and a logical reasoning is that being a consequence of unattributed peaks that could no longer be explained by drop-in. In both cases, in mixtures with two and three estimated contributors, the maximal computed differences were much bigger for STRmix™ values than those from EuroForMix. Nevertheless, and as previously referred, the large differences obtained in STRmix™ analyses raised the hypothesis of these being a consequence of the stochasticity of the model under which the software is based, rather than of the different models themselves. Indeed, STRmix™ assigns a relative weight to the probability of the EPG given each possible genotype configuration using MCMC methods, which implies, by definition, that the results of different runs will not coincide. Nevertheless, small variations in comparison to the LR value itself, with minimal practical impact, were expected. The hypothesis of the lack of convergence was thus further explored considering the genotypic data of the sample pair 13_2, for which the maximum difference R was observed. Under the same conditions, computations were replicated three times for default and varied parameters, and very different results were obtained between trials—see Supplementary Table S4. These results support that the great differences observed may be consequence of a lack of convergence of the model that can be solved by increasing burn-in and post burn-in accepts at the expense of runtime and computer capacity costs.

### Analytical threshold

Table 4 shows the impact on the LR value of increasing the analytical threshold from 100 RFU (default value) to 150 RFU (varied value), for both mixtures with two and three estimated contributors in quantitative tools. A possible trend for the LR increasing or decreasing when the analytical threshold was varied is also presented.

**Table 4:** Ratio R (R > 1) between LR values computed through EuroForMix v.3.4.0, and STRmix™ v.2.7, considering the analytical threshold equal to 100 RFU (default) and 150 RFU (varied), for mixtures with two and three estimated contributors.

Ratio between LRs (100 RFU vs 150 RFU, R > 1)	Estimated number of contributors			
	2		3	
	EuroForMix	STRmix™	EuroForMix	STRmix™
1 < R < 10	81%	92%	87%	87%
10 < R < 100	16%	6%	6%	8%
100 < R < 1000	1%	0%	4%	1%
1000 < R < 10,000	1%	0%	1%	1%
R > 10,000	1%	1%	1%	3%
Maximum ratio between LRs				
R	9.44E+04	2.41E+06	3.36E+04	1.37E+11
LR (default)	8.21E+18	3.80E+09	3.46E+07	1.51E+04
LR (varied)	8.70E+13	1.58E+03	1.03E+03	1.10E−07
Pair*	25_2	13_2	11_3	9_3
LR tends to (α = 0.05)				
	Decrease	–	Decrease	–
p-value	2.09E−03	7.32E−01	4.11E−04	7.32E−01

The evidence of a general trend for the LR values increasing or decreasing when the analytical threshold was increased is presented along with the corresponding p-value (α = 0.05); significant values are underlined. *See Tables S3_3 in Supplementary Table S3.

No statistical evidence was found regarding a possible general trend to the decrease or increase of the LR values computed by STRmix™. On the other hand, a statistically significant trend for EuroForMix LR values decrease when the analytical threshold value increased was verified for both cases with mixtures with two and three estimated contributors (Table 4).

For most cases, the difference between the two LR values computed does not overpass ten times. However, differences higher than 10,000 times were found. In almost all cases where differences higher than 10,000 times were reached, a decrease in the LR value occurred. This decrease was determined by alleles that matched the reference sample but were below the analytical threshold—as it happens in EuroForMix—and/or by the change in the allele peak height—as it happens in STRmix™. In only one case, with a difference higher than 10,000 times, the LR value increased when the analytical threshold value was increased. This situation occurred for STRmix™ and resulted from the non-consideration of alleles matching the other possible contributors of the mixture, as they were below the analytical threshold considered. Nevertheless, and as previously referred to and shown, caution should be taken when assuming parameter variation as the main source of different LR values when considering a software based on stochastic methods as is the case of STRmix™ as these can be also due to the lack of convergence of the model.

### Stutter artifacts

LR values computed using EuroForMix software were compared, assuming the presence and absence of stutters, for both cases with mixtures with two and three estimated contributors. The differences found between those values are presented in Table 5, as well as an analysis regarding a possible general trend for the LR increase or decrease.

**Table 5:** Ratio R (R > 1) between LR values computed through EuroForMix v.3.4.0 with (default) and without stutters (varied condition), for mixtures with two and three estimated contributors.

Ratio between LRs (with vs without stutters, R > 1)	Estimated number of contributors	
	2	3
1 < R < 10	74%	78%
10 < R < 100	17%	13%
100 < R < 1000	5%	3%
1000 < R < 10,000	1%	5%
R > 10,000	3%	1%
Maximum ratio between LRs		
R	6.91E+05	3.68E+05
LR (default)	1.41E+10	1.65E+10
LR (varied)	9.75E+15	6.07E+15
Pair*	16_2	9_3
LR tends to (α = 0.05)		
	Decrease	Decrease
p-value	4.39E−03	9.50E−04

The evidence of a general trend for the LR values increase or decrease when the stutters were not considered is presented along with the corresponding p-value (α = 0.05); significant values are underlined. *See Tables S3_4 in Supplementary Table S3.

A stutter peak is a PCR product artifact formed during the PCR extension phase, as previously mentioned. So, it can be said that the product from an allele amplification is a combination of correct copies of the target allele and the corresponding stutter peaks. For this reason, the LR value should tend to increase when the stutters’ peaks are included, as confirmed by our data for which the reference sample cannot be excluded as a contributor to problem one.

As demonstrated in Table 5, most cases do not reach differences greater than ten times. Nevertheless, differences of more than 10,000 times were computed, reaching the maximum value of 691,489 times.

## Conclusions

The ambition to analyze increasingly complex samples, namely DNA mixture samples, led to the development of software tools to support the expert in the interpretation of the results. These tools apply mathematical models implied in the computation of a probative value to quantify the genetic evidence. This calculation involves the setting of several parameters regarding population genetics and analytical factors, which are estimated either directly by the software or introduced by the user.

Therefore, it is fundamental to demonstrate the impact on statistical evaluation of the genetic evidence when considering different values and/or conditions in the software parameters. So, this work aims to highlight the importance of properly and accurately estimating and modeling parameters such as drop-in, analytical threshold, and stutters, for forensic genetics routine analyses.

LR values were computed under the established default and varied conditions, varying a single parameter at a time, for each one of 154 real casework mixture/single-source sample pairs, the first with two or three estimated contributors. The results obtained through different informatic tools—one qualitative (LRmix Studio v.2.1.3), and two quantitative (EuroForMix v.3.4.0, and STRmix™ v.2.7) for 21 independent and autosomal STRs, were compared and the impact of parameters’ variation within and between software was evaluated.

Even though the proportion of cases differing more than 10,000 times was low for each varied parameter, the modest dataset size (77 mixtures with two estimated contributors, and 77 with three) and the magnitude of differences reached imply that the impact of these parameters on the weight of the evidence should not be overlooked, especially for cases where considering different values and/or conditions resulted in the support of opposite hypotheses. In this regard, it should however be noted that the stochasticity of the methods grounding one of the informatic tools used: STRmix™, may hamper the corresponding conclusions as the observed differences may be mostly a consequence of a lack of convergence of the MCMC algorithms rather than of the parameter variation itself. Indeed, this was the case for the maximum difference found—LR computed assuming the default condition regarding drop-in modeling in STRmix™ (Gamma distribution γ = (1.0, 1.0), LR = 3.80E+09) that differed 2.12E+17 times from the LR computed assuming the varied condition (Uniform distribution, LR = 1.79E−08), each one supporting opposite hypotheses. Maintaining the same MCMC parameter values, computations were replicated three more times for default and varied conditions, the variation of the results greatly differing from those obtained in the first trial. The lack of convergence is due to sample-specific factors that affect precision and can be solved by running the problem for longer, i.e. for more MCMC accepts.

Focusing on the Drop-in parameter, in the qualitative software (LRmix Studio) a statistically significant trend for the LR value decrease with increasing drop-in frequency was observed, but none of the calculated differences exceeded 10,000 times. The same trend was observed for EuroForMix. Discrepancies greater than 10,000 times were verified for both quantitative software, varying between 0 and 3% when the frequency value was tested, and also when the distribution model was varied. Nevertheless, and again, the possibility of some of these differences being also a result of the stochastic method on which STRmix™ is based should not be disregarded.

Regarding the analytical threshold parameter, evaluated in quantitative software, only for the LR values computed in EuroForMix was observed a statistically significant trend: when the analytical threshold was increased from 100 to 150 RFU, the retrieved EuroForMix LR values generally decreased. The proportion of cases where the LR differed more than 10,000 times remained at 1% for EuroForMix (for both cases with mixtures with two and three estimated contributors), while for STRmix™ varied between 1 and 3%, respectively. Yet, as previously mentioned, caution should be taken when analyzing STRmix™ LR values, as the source of variation may not be just a result of considering different parameter conditions.

Finally, when considering stutters (only evaluated in EuroForMix), computed LR values were shown to be higher when stutters were considered. Differences greater than 10,000 times were found in both cases with mixtures with two (3%) and three (1%) estimated contributors.

Thus, another step is added to the important role of the experts when they are working with probabilistic genotyping programs. Before performing any computation, the expert must establish and introduce the values determined either empirically following internal protocols or based on values presented in the literature, for each parameter of the software, so that the computation of the LR value is as accurate as possible. The analysis of the unknown sample profile EPG, the evaluation of computed LR value, and the estimation of software parameter values depend on the expert and constitute crucial steps that may influence the interpretation of the results if wrongly or inaccurately performed. Indeed, this work emphasizes the importance of proper modeling and estimation of the parameters that influence the weight of the evidence, along with appropriate training and awareness of the experts on the mathematical/statistical models implemented in the probabilistic genotyping tools that have been introduced in the forensic routine. Special attention should be devoted when computer tools based on stochastic methodologies are used, for which, ideally, different number of runs to testify the convergence of the algorithms should be performed. Indeed, testing the reproducibility and repeatability of informatics tools is a relevant and important topic that should be further investigated in the future. This is particularly pertinent in the forensic genetics setting where methodologies have been based, at least theoretically, in completely reproducible interpretation results, being the application of stochastic systems as MCMC relatively new in the field.

The parameter settings considered in this work were established to demonstrate the impact that different values may have in the quantification of the evidence using different informatics and statistical approaches. Despite being within a reasonable range for most situations, the considered values cannot be seen, by any means, as suggested values. Each laboratory should estimate and establish their own parameter settings, considering their conditions and specificities, using appropriate, dedicated, and up-to-date methodologies.

In summary, this work reinforces and alerts all practitioners to the importance of considering and accurately estimating the laboratory-specific values/conditions for all the parameters mentioned, underlining the need for proper user trainning and laboratory-specific validation of any software prior to its use.

## Supplementary Information

Supplementary Information 1.

Supplementary Information 2.

Supplementary Information 3.

Supplementary Information 4.

## Data Availability

All the statistics that supported the results presented in this study are provided in Supplementary Material, for each one of the analyzed pairs of samples and parameters. The raw genotypic and haplotypic analyzed data concern real forensic casework from the Portuguese Scientific Police Laboratory, Judiciary Police (LPC-PJ) and for that reason, its release is not possible. In any case, specific data from specific analyzed markers (non-haplotypic data) may be available from the authors upon reasonable request and with the permission of LPC-PJ. For data sharing issues contact the corresponding author of the manuscript.

## References

[1] Brownstein, M., Carpten, J. & Smith, J. Modulation of non-templated nucleotide addition by TaqDNA polymerase: primer modifications that facilitate genotyping. *Biotechniques* **20**(4), 1004–1010 (1996). 8780871 10.2144/96206st01

[2] Magnuson, V. et al. Substrate nucleotide-determined non-templated addition of adenine by TaqDNA polymerase: implications for PCR-based genotyping and cloning. *Biotechniques* **21**(4), 700–709 (1996). 8891224 10.2144/96214rr03

[3] Walsh, P., Fildes, N. & Reynolds, R. Sequence analysis and characterization of stutter products at the tetranucleotide repeat locus vWA. *Nucleic Acids Res.* **24**, 2807–2812 (1996). 8759015 10.1093/nar/24.14.2807 PMC146018

[4] Gibb, A. J., Huell, A. L., Simmons, M. C. & Brown, R. M. Characterisation of forward stutter in the AmpFlSTR SGM Plus PCR. *Sci. Justice* **49**(1), 24–31 (2009). 19418925 10.1016/j.scijus.2008.05.002

[5] Dash, H., Shivastava, P. & Das, S. Analysis of capillary electrophoresis results by GeneMapper^®^ ID-X v 1.5 software. In *Principles and Practices of DNA Analysis: A Laboratory Manual for Forensic DNA Typing.* (Springer Protocols Handbooks, Humana, 2020).

[6] Westen, A. A. et al. Higher capillary electrophoresis injection settings as an efficient approach to increase the sensitivity of STR typing. *J. Forensic Sci.* **54**(3), 591–598 (2009). 19432738 10.1111/j.1556-4029.2009.01022.x

[7] Balding, D. J. & Buckleton, J. Interpreting low template DNA profiles. *Forensic Sci. Int. Genet.* **4**(1), 1–10 (2009). 19948328 10.1016/j.fsigen.2009.03.003

[8] Gittelson, S., Biedermann, A., Bozza, S. & Taroni, F. Decision analysis for the genotype designation in low-template-DNA profiles. *Forensic Sci. Int. Genet.* **9**, 118–133 (2014). 24528590 10.1016/j.fsigen.2013.11.005

[9] Steele, C. D., Greenhalgh, M. & Balding, D. J. Evaluation of low-template DNA profiles using peak heights. *Stat. Appl. Genet. Mol. Biol.* **15**(5), 431–445 (2016). 27416618 10.1515/sagmb-2016-0038

[10] Butler, J. M. STR alleles and amplification artifacts. In *Advanced Topics in Forensic DNA Typing: Interpretation.* 47–86 (2015).

[11] Clayton, T., Whitaker, J., Sparkes, R. & Gill, P. Analysis and interpretation of mixed forensic stains using DNA STR profiling. *Forensic Sci. Int.* **91**(1), 55–70 (1998). 9493345 10.1016/s0379-0738(97)00175-8

[12] Methods SWGoDA: SWGDAM interpretation guidelines for autosomal STR typing by forensic DNA testing laboratories. (2010).

[13] Butler, J. M. *Advanced Topics in Forensic DNA Typing: Interpretation* (Academic Press, 2014).

[14] Gill, P. *Forensic Practitioner’s Guide to the Interpretation of Complex DNA Profiles* (Academic Press, 2020).

[15] Norsworthy, S., Lun, D. S. & Grgicak, C. M. Determining the number of contributors to DNA mixtures in the low-template regime: exploring the impacts of sampling and detection effects. *Legal Med.* **32**, 1–8 (2018). 29453054 10.1016/j.legalmed.2018.02.001

[16] Lauritzen, S. L. & Mortera, J. Bounding the number of contributors to mixed DNA stains. *Forensic Sci. Int.* **130**(2–3), 125–126 (2002). 12477632 10.1016/s0379-0738(02)00351-1

[17] Costa, C. et al. Quantification of forensic genetic evidence: Comparison of results obtained by qualitative and quantitative software for real casework samples. *Forensic Sci. Int. Genet.* **59**, 102715 (2022). 35490558 10.1016/j.fsigen.2022.102715

[18] Lee, H. & Ladd, C. Preservation and collection of biological evidence. *Croat. Med. J.* **42**(3), 225–228 (2001). 11387627

[19] Sheppard, K., Cassella, J. P., Fieldhouse, S. & King, R. The adaptation of a 360 degrees camera utilising an alternate light source (ALS) for the detection of biological fluids at crime scenes. *Sci. Justice* **57**(4), 239–249 (2017). 28606329 10.1016/j.scijus.2017.04.004

[20] Malegori, C. et al. Identification of invisible biological traces in forensic evidences by hyperspectral NIR imaging combined with chemometrics. *Talanta* **215**, 120911 (2020). 32312455 10.1016/j.talanta.2020.120911

[21] Taylor, D. et al. Inter-sample contamination detection using mixture deconvolution comparison. *Forensic Sci. Int. Genet.* **40**, 160–167 (2019). 30851600 10.1016/j.fsigen.2019.02.021

[22] Kloosterman, A., Sjerps, M. & Quak, A. Error rates in forensic DNA analysis: definition, numbers, impact and communication. *Forensic Sci. Int. Genet.* **12**, 77–85 (2014). 24905336 10.1016/j.fsigen.2014.04.014

[23] Haned, H., Gill, P., Lohmueller, K., Inman, K. & Rudin, N. Validation of probabilistic genotyping software for use in forensic DNA casework: definitions and illustrations. *Sci. Justice* **56**(2), 104–108 (2016). 26976468 10.1016/j.scijus.2015.11.007

[24] Steele, C. D. & Balding, D. J. Statistical evaluation of forensic DNA profile evidence. *Annu. Rev. Stat. Appl.* **1**, 361–384 (2014).

[25] Gill, P. et al. DNA commission of the International Society of Forensic Genetics: Recommendations on the interpretation of mixtures. *Forensic Sci. Int.* **160**(2–3), 90–101 (2006). 16750605 10.1016/j.forsciint.2006.04.009

[26] Slooten, K. Accurate assessment of the weight of evidence for DNA mixtures by integrating the likelihood ratio. *Forensic Sci. Int. Genet.* **27**, 1–16 (2017). 27914277 10.1016/j.fsigen.2016.11.001

[27] Alladio, E. et al. DNA mixtures interpretation—A proof-of-concept multi-software comparison highlighting different probabilistic methods’ performances on challenging samples. *Forensic Sci. Int. Genet.* **37**, 143–150 (2018). 30173123 10.1016/j.fsigen.2018.08.002

[28] Coble, M. D. & Bright, J. A. Probabilistic genotyping software: An overview. *Forensic Sci. Int. Genet.* **38**, 219–224 (2019). 30458407 10.1016/j.fsigen.2018.11.009

[29] Inman, K. et al. Lab Retriever: a software tool for calculating likelihood ratios incorporating a probability of drop-out for forensic DNA profiles. *BMC Bioinform.* **16**, 298 (2015). 10.1186/s12859-015-0740-8 PMC4575494 26384762

[30] Haned, H., Slooten, K. & Gill, P. Exploratory data analysis for the interpretation of low template DNA mixtures. *Forensic Sci. Int. Genet.* **6**(6), 762–774 (2012). 22981542 10.1016/j.fsigen.2012.08.008

[31] Bleka, O., Storvik, G. & Gill, P. EuroForMix: An open source software based on a continuous model to evaluate STR DNA profiles from a mixture of contributors with artefacts. *Forensic Sci. Int. Genet.* **21**, 35–44 (2016). 26720812 10.1016/j.fsigen.2015.11.008

[32] Taylor, D., Bright, J. A. & Buckleton, J. The interpretation of single source and mixed DNA profiles. *Forensic Sci. Int. Genet.* **7**(5), 516–528 (2013). 23948322 10.1016/j.fsigen.2013.05.011

[33] Bille, T. W., Weitz, S. M., Coble, M. D., Buckleton, J. & Bright, J. A. Comparison of the performance of different models for the interpretation of low level mixed DNA profiles. *Electrophoresis* **35**(21–22), 3125–3133 (2014). 25168355 10.1002/elps.201400110

[34] Bleka, Ø., Benschop, C. C., Storvik, G. & Gill, P. A comparative study of qualitative and quantitative models used to interpret complex STR DNA profiles. *Forensic Sci. Int. Genet.* **25**, 85–96 (2016). 27529774 10.1016/j.fsigen.2016.07.016

[35] Alladio, E. et al. DNA mixtures interpretation–a proof-of-concept multi-software comparison highlighting different probabilistic methods’ performances on challenging samples. *Forensic Sci. Int. Genet.* **37**, 143–150 (2018). 30173123 10.1016/j.fsigen.2018.08.002

[36] Riman, S., Iyer, H. & Vallone, P. M. Examining performance and likelihood ratios for two likelihood ratio systems using the PROVEDIt dataset. *PLoS ONE* **16**(9), e0256714 (2021). 34534241 10.1371/journal.pone.0256714 PMC8448353

[37] Boodoosingh, S., Kelly, H., Curran, J. M. & Kalafut, T. An inter-laboratory comparison of probabilistic genotyping parameters and evaluation of performance on DNA mixtures from different laboratories. *Forensic Sci. Int. Genet.* **71**, 103046 (2024). 38598920 10.1016/j.fsigen.2024.103046

[38] Gill, P., Whitaker, J., Flaxman, C., Brown, N. & Buckleton, J. An investigation of the rigor of interpretation rules for STRs derived from less than 100 pg of DNA. *Forensic Sci. Int.* **112**(1), 17–40 (2000). 10882828 10.1016/s0379-0738(00)00158-4

[39] Gill, P., Kirkham, A. & Curran, J. LoComatioN: a software tool for the analysis of low copy number DNA profiles. *Forensic Sci. Int.* **166**(2–3), 128–138 (2007). 16759831 10.1016/j.forsciint.2006.04.016

[40] Tvedebrink, T., Eriksen, P. S., Mogensen, H. S. & Morling, N. Estimating the probability of allelic drop-out of STR alleles in forensic genetics. *Forensic Sci. Int. Genet.* **3**(4), 222–226 (2009). 19647706 10.1016/j.fsigen.2009.02.002

[41] Buckleton, J. et al. Utilising allelic dropout probabilities estimated by logistic regression in casework. *Forensic Sci. Int. Genet.* **9**, 9–11 (2014). 24528573 10.1016/j.fsigen.2013.07.001

[42] Puch-Solis, R. A dropin peak height model. *Forensic Sci. Int. Genet.* **11**, 80–84 (2014). 24657454 10.1016/j.fsigen.2014.02.005

[43] Kelly, H., Bright, J. A., Coble, M. D. & Buckleton, J. S. A description of the likelihood ratios in the probabilistic genotyping software STRmix™. *WIREs Forensic Sci.* 2(6) (2020).

[44] Cheng, K. et al. A comparison of likelihood ratios obtained from EuroForMix and STRmix™. *J. Forensic Sci.* **66**(6), 2138–2155 (2021). 34553371 10.1111/1556-4029.14886

[45] Haned, H., Egeland, T., Pontier, D., Pene, L. & Gill, P. Estimating drop-out probabilities in forensic DNA samples: a simulation approach to evaluate different models. *Forensic Sci. Int. Genet.* **5**(5), 525–531 (2011). 21216685 10.1016/j.fsigen.2010.12.002

[46] Mitchell, A. A. et al. Likelihood ratio statistics for DNA mixtures allowing for drop-out and drop-in. *Forensic Sci. Int. Genet. Suppl. Ser.* **3**(1), e240–e241 (2011).

[47] Mitchell, A. A. et al. Validation of a DNA mixture statistics tool incorporating allelic drop-out and drop-in. *Forensic Sci. Int. Genet.* **6**(6), 749–761 (2012). 22999739 10.1016/j.fsigen.2012.08.007

[48] Bregu, J. et al. Analytical thresholds and sensitivity: establishing RFU thresholds for forensic DNA analysis. *J. Forensic Sci.* **58**(1), 120–129 (2013). 23130820 10.1111/1556-4029.12008

[49] Hansson, O., Gill, P. & Egeland, T. STR-validator: an open source platform for validation and process control. *Forensic Sci. Int. Genet.* **13**, 154–166 (2014). 25178680 10.1016/j.fsigen.2014.07.009

[50] Bright, J.-A., Taylor, D., Curran, J. M. & Buckleton, J. S. Developing allelic and stutter peak height models for a continuous method of DNA interpretation. *Forensic Sci. Int. Genet.* **7**(2), 296–304 (2013). 23317914 10.1016/j.fsigen.2012.11.013

[51] Bleka, Ø. & Gill, P. Interpretation of a complex STR DNA profile using EuroForMix. *Forensic Sci. Int. Genet. Suppl. Ser.* **5**, e405–e406 (2015).

[52] Weir, B. S. The second National Research Council report on forensic DNA evidence. *Am. J. Hum. Genet.* **59**(3), 497 (1996). 8751849 PMC1914912

[53] Hill, C. R., Duewer, D. L., Kline, M. C., Coble, M. D. & Butler, J. M. US population data for 29 autosomal STR loci. *Forensic Sci. Int. Genet.* **7**(3), e82–e83 (2013). 23317915 10.1016/j.fsigen.2012.12.004

[54] Moretti, T. R. et al. Internal validation of STRmix™ for the interpretation of single source and mixed DNA profiles. *Forensic Sci. Int. Genet.* **29**, 126–144 (2017). 28504203 10.1016/j.fsigen.2017.04.004

[55] Haned, H., Benschop, C. C., Gill, P. D. & Sijen, T. Complex DNA mixture analysis in a forensic context: evaluating the probative value using a likelihood ratio model. *Forensic Sci. Int. Genet.* **16**, 17–25 (2015). 25485478 10.1016/j.fsigen.2014.11.014

[56] Costa, C. et al. Statistical analysis tools of mixture DNA samples: When the same software provides different results. *Forensic Sci. Int. Genet. Suppl. Ser.* **8**, 37–39 (2022).

