# Supplementary material for: The impact of parameter variation in the quantification of forensic genetic evidence
Source: Sci Rep. 2025 Jan 20;15:2524. doi: 10.1038/s41598-024-83841-2 (PMC11756398; doi:10.1038/s41598-024-83841-2)
Supplement: Supplementary file 2 — Supplementary Information 2. [file 41598_2024_83841_MOESM2_ESM.pdf]

Table S2: Parameter values considered in LRmix Studio v.2.1.3, EuroForMix v.3.4.0, and STRmix™ v.2.7 during the computation of the LRs analyzed in this work, to weigh the parameters' variation impact in LR computation.

**Data**	**Parameters values**				**Analyzed Parameter**			
	**Analytical Threshold**	**Drop-in**		**Stutters**	**Analytical Threshold**	**Drop-in**		**Stutters**
		**Frequency**	**Parameters' distribution**			**Frequency**	**Parameters' distribution**	
**EuroForMix v.3.4.0**	100	0.05	λ = 0.01	Yes	X	X	X	X
	100	0.05	λ = 0.01	**No**				X
	**150**	0.05	λ = 0.01	Yes	X			
	100	**0.00**	λ = 0.01	Yes		X		
	100	**0.10**	λ = 0.01	Yes		X		
	100	0.05	**λ = 0.05**	Yes			X	
**STRmix ™ v.2.7**	100	0.05	ɣ = 1.0, 1.0	Yes	X	X	X	
	**150**	0.05	ɣ = 1.0, 1.0	Yes	X			
	100	**0.00**	ɣ = 1.0, 1.0	Yes		X		
	100	**0.10**	ɣ = 1.0, 1.0	Yes		X		
	100	0.05	**Uniform**	Yes			X	
**LRmix Studio v .2.1.3**	-	0.05	-	No		X		
	-	**0.00**	-	No		X		
	-	**0.10**	-	No		X		
